# mRNA Processing: An Emerging Frontier in the Regulation of Pancreatic β Cell Function

**DOI:** 10.3389/fgene.2020.00983

**Published:** 2020-09-01

**Authors:** Nicole D. Moss, Lori Sussel

**Affiliations:** Cell, Stem Cells, and Development Graduate Program, Department of Pediatrics, Barbara Davis Center, University of Colorado Denver Anschutz Medical Campus, Aurora, CO, United States

**Keywords:** pancreatic islet, beta cells, diabetes, RNA processing, RNA binding proteins

## Abstract

Robust endocrine cell function, particularly β cell function, is required to maintain blood glucose homeostasis. Diabetes can result from the loss or dysfunction of β cells. Despite decades of clinical and basic research, the precise regulation of β cell function and pathogenesis in diabetes remains incompletely understood. In this review, we highlight RNA processing of mRNAs as a rapidly emerging mechanism regulating β cell function and survival. RNA-binding proteins (RBPs) and RNA modifications are primed to be the next frontier to explain many of the poorly understood molecular processes that regulate β cell formation and function, and provide an exciting potential for the development of novel therapeutics. Here we outline the current understanding of β cell specific functions of several characterized RBPs, alternative splicing events, and transcriptome wide changes in RNA methylation. We also highlight several RBPs that are dysregulated in both Type 1 and Type 2 diabetes, and discuss remaining knowledge gaps in the field.

## Introduction

**FIGURE 1:**
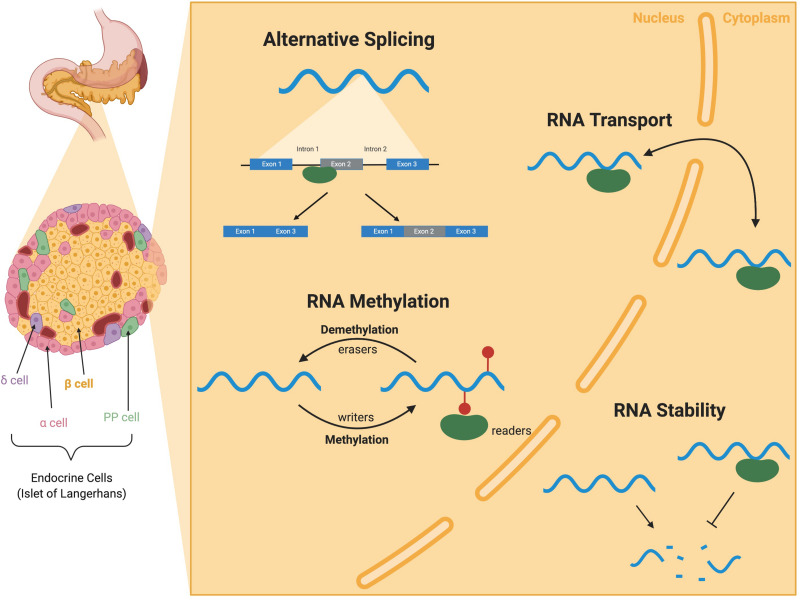
*RNA-binding protein (RBP) mediated RNA regulation in the pancreatic* β *cell.* I nsulin-secreting β cells reside in the islets of the pancreas along with several other endocrine cell types (α, δ, and PP cells). RBPs (green) are present in both the nucleus and the cytoplasm of cells and bind to RNA (blue) to perform a variety of functions. RBPs binding to introns and exons of pre-mRNAs contribute to *alternative splicing*. RBPs can also write, read, and erase methylation modification on mRNAs in *RNA methylation.* RBPs can also facilitate the *transport* of RNAs between the nucleus and cytoplasm and throughout the cell. RBP binding to the UTRs can alter mRNA stability and translation. Illustration created with BioRender.

The highly specialized insulin-producing β cell population is located within the pancreatic islet of Langerhans (Figure 1). In humans and other vertebrates, β cells respond to changes in circulating blood glucose levels by secreting insulin. Coupled with the function of the other islet endocrine cell types, β cells help to maintain blood glucose homeostasis; loss or dysfunction of the β cell population results in diabetes. Over the last several decades, substantial research efforts have been directed toward understanding the gene regulatory networks required for the formation and function of the islet cell populations. This has included developmental studies in model organisms that have identified the key transcription factors required to make and maintain functional β cell populations. In addition, translational research approaches in human subjects, including Genome Wide Association Studies (GWAS) and other large sequencing efforts, have identified numerous alleles and mutations associated with increased risk of developing either Type 1 (T1D) or Type 2 (T2D) diabetes, many of which cause β cell dysfunction. Despite these efforts, there remain many gaps in our understanding of the mechanisms that regulate β cells and the pathways that contribute to their pathogenesis in diabetes.

Although much of the research to date has been focused on transcriptional regulation, β cell identity and function are also regulated at the level of mRNA, similar to many other cell types and organ systems. Throughout their life cycle, mRNA molecules undergo extensive processing events to transition from a pre-mRNA molecule to a mature mRNA. These events not only include addition of a 5′-cap and 3′-poly-A tail, but also splicing of introns, nucleotide modification, stability, and subcellular localization (Licatalosi and Darnell, 2010; Figure 1). RNA-binding proteins (RBPs) are responsible for coordinating the events in the lifecycle of an mRNA. Over the past few years, several groups have begun to probe the function of specific RBPs in organogenesis and disease. Many studies have focused on the mis-regulation of mRNAs and RBP function in the context of diabetic complications (adipose, liver, muscle, retina, etc.), rather than specific changes in the β cells (Nutter and Kuyumcu-Martinez, 2018). In the pancreas, only a few groups have delved into the world of RNA regulation, often focusing on a single splicing target or RBP. In this review, we will highlight these studies describing RBP functions, transcriptome wide changes in RBP expression, alternative splicing, and RNA methylation, with a specific focus on regulation of mRNAs in the pancreatic islet population. This is a rapidly emerging field that will undoubtedly provide a unique perspective on a complex disease and will ultimately push the boundaries of therapeutic treatments for diabetes.

### RNA-Binding Proteins in the β Cell

Several hundred RBPs have been identified (Hentze et al., 2018), each with the potential of having hundreds of targets within a cell (Keene, 2007; Hogan et al., 2008; Lukong et al., 2008; Blanchette et al., 2009; Li et al., 2014). Some RBPs have ubiquitous expression, while others are transiently expressed during development or restricted to a specific cell type (Gerstberger et al., 2014). Like many other proteins, RBPs are categorized by several modular domains. RBPs recognize RNA targets through a binding domain, in the form of an RNA recognition motif (RRM), K-homology (KH) domain, and RNA-binding zinc-finger (ZnF) domains, or can bind independent of sequence through a double-stranded RNA module (dsRBD) (Lunde et al., 2007). Additionally, RBPs have a variety of enzymatic and/or signaling domains that allow for functional activity (Lunde et al., 2007).

The role of RBPs in the formation and function of pancreatic endocrine cells is only beginning to be appreciated. Only a small number of known RBPs have been studied in the β cell, but as new transcriptomics data becomes available from both healthy and diseased islets, their role in β cell biology will become more apparent. Recently, several studies have identified RBPs that are enriched in pancreatic islet cells and become dysregulated under stress (Juan-Mateu et al., 2017; Jeffery et al., 2019; Ramos-Rodriguez et al., 2019). Stressors including chronic hyperglycemia (Puri and Hebrok, 2012; Brereton et al., 2014), exposure to pro-inflammatory cytokines (Ortis et al., 2010), and palmitate (saturated fatty acid) (Cnop et al., 2014) can result in changes in cellular and molecular identity. In a model of human β cells (EndoC-βH1), treatment with cell stressors (including cytokines, hypoxia, altered lipids, and high and low levels of glucose) also induced dysregulation of many RBPs (Jeffery et al., 2019).

Islet endocrine cells have a specific assemblage of RBPs that perform a variety of functions. Re-analysis of whole transcriptomic RNA-Sequencing (RNA-Seq) data from several human tissues (Eizirik et al., 2012) revealed that human islets share a notable number of RBPs with the brain, and β cells in particular are enriched for many “neuron specific” RBPs (Juan-Mateu et al., 2017; Alvelos et al., 2018). This is not surprising since, despite their disparate developmental origins, neurons and β cells share a large number of transcriptional networks (van Arensbergen et al., 2010). While it is clear that there are a whole host of RBPs expressed in mature insulin-secreting β cells (summarized in Table 1), there remains poor understanding about the role of RBP regulation in the developing pancreas (Baralle and Giudice, 2017). Additionally, the majority of these studies to date are limited to *in vitro* analysis of RBP requirements and molecular function. RBPs regulate several classes of RNAs, including both coding (mRNA) and non-coding (ncRNA) RNAs. A recent comprehensive review has discussed the critical role of RBPs Dicer and Argonaut in the regulation of miRNAs for β cell function and in T2D (Eliasson and Esguerra, 2020). Here we will provide a brief survey of the expression and function of several prominent RBP families that regulate mRNAs in pancreatic β cells.

**TABLE 1:** RNA-Binding Proteins (RBPs) in the Pancreatic β Cell.

RBP	Dysregulated condition	β cell specific function	References
AKAP17A	Dysregulated under cytokine treatment	*NA*	Jeffery et al., 2019
AUF1 (hnRNP-D)	Cytokine treatment reduces nuclear AUF1 without decreases in total AUF1	Increased AUF1 promotes apoptosis	Roggli et al., 2012; Vanzela and Cardozo, 2012; Magro and Solimena, 2013
CELF1/CUGBP1	Increased expression in diabetic models	Decreased GSIS by stabilizing PDE3B mRNA which mediates cAMP hydrolysis	Zhai et al., 2016; Nutter and Kuyumcu-Martinez, 2018; Good and Stoffers, 2020
DDX1	Decreased function under lipotoxicity	Regulates alternative splicing of voltage gaited Ca^2 +^ channels and increases insulin translation through interactions translation initiation factors	Li et al., 2018; Zhong et al., 2018; Good and Stoffers, 2020
FTO	Decreased expression in T2D islets	Controversial regulation of insulin secretion	Kirkpatrick et al., 2010; Russell and Morgan, 2011; Dayeh et al., 2014; Fan et al., 2015; Taneera et al., 2015, 2018
hnRNPA2B1	Dysregulated under both high and low glucose conditions, hypoxia, and cytokine treatment	*NA*	Jeffery et al., 2019
hnRNPK/DDX3X	hnRNPK is phosphorylated and activated under metabolic stress	Binds to JUND 3′UTR to regulate translation	Good et al., 2019; Good and Stoffers, 2020
HuD (ELAVL4)	ER stress increases expression, HuD expression is glucose responsive and reduced in diabetes	Increased nuclear HuD results in decreased insulin biosynthesis, binds the 5′UTR of *Ins2* mRNA and decreases *Ins2* translation, regulates ATG5 translation and Mnf2 stability	Lee et al., 2012; Magro and Solimena, 2013; Yoo, 2013; Kim et al., 2014; Juan-Mateu et al., 2017; Hong et al., 2020
IMP	IMP3 dysregulated under lipotoxicity, IMP2 SNPs associated with moderately increased risk of T2D	*NA*	Christiansen et al., 2009; Nutter and Kuyumcu-Martinez, 2018; Jeffery et al., 2019
LSM14A	Dysregulated expression under low glucose and cytokine treatment	*NA*	Jeffery et al., 2019
Mushashi 1/2	ER stress increases expression of *Msi1* and *Msi2* and lipotoxicity increases expression of *Msi2*	Musashi 1 regulates ββ cell proliferation and both Musashi 1 and 2 decrease insulin gene expression	Szabat et al., 2011; Magro and Solimena, 2013
Nova 1/2	Decreased expression in cytokine treated cells	Loss of NOVA1 results in decreased insulin secretion and loss of either NOVA1 or NOVA2 results in decreased apoptosis	Villate et al., 2014; Juan-Mateu et al., 2017
PDI/PABP	*NA*	PDI binds the 5′UTR of insulin mRNA to promote insulin biosynthesis through interactions with PABP, PDI/PABP associate with insulin, PC1/3, and PC2 5′UTR to regulate translation, PABP can also interact with HuD to suppress insulin translation	Kulkarni et al., 2011; Magro and Solimena, 2013; Sarwade et al., 2020
PNISR	Dysregulated expression under low glucose, hypoxia, and cytokine treatment	*NA*	Jeffery et al., 2019
PTBP1 (hnRNP1/PTB)	hypoxia and prolonged high glucose leads to decreased PTB1 expression	PTB binds both insulin mRNA and insulin granule proteins to regulate stability and translation	Tillmar et al., 2002; Tillmar and Welsh, 2002; Knoch et al., 2004; Knoch et al., 2006; Fred et al., 2010, 2011, 2016; Magro and Solimena, 2013
Rbfox	*NA*	Rbfox1 and Rbfox2 modulate insulin secretion by regulating actin modifying proteins	Juan-Mateu et al., 2017; Good and Stoffers, 2020
RBM4	*NA*	Regulates alternative splicing of key β cell transcription factors (*Isl1, Pax4, Pax6, Glut2)*	Lin et al., 2013; Magro and Solimena, 2013
SRSF 1/2/3/6	SRSF1/3/6 are dysregulated under low glucose, SRSF3/6 are dysregulated under hypoxia, SRSF1/2/3 dysregulated in response to cytokine treatment	NA	Jeffery et al., 2019

#### Hu and Embryonic Lethal Abnormal Vision-Like Protein Family (HuD/ELAVL4)

The Hu/ELAV family of RBPs bind to AU-rich elements (AREs) in the 3′UTR of mRNAs and can modulate transcript stability and translation (Hinman and Lou, 2008). Hu/ELAV proteins bind to the AREs through three RRMs (Okano and Darnell, 1997). HuR/ELAVL1 is ubiquitously expressed, while the other three family members (HuB/ELAVL2, HuC/ELAVL3, HuD/ELAVL4) are most highly expressed in neurons (Hinman and Lou, 2008). However, a few recent studies have identified roles for one of these family members, HuD/ELAVL4, in the β cell (Lee et al., 2012; Kim et al., 2014; Hong et al., 2020). Normally, HuD expression is (1) glucose dependent; (2) regulated through insulin receptor (INSR) signaling; and (3) acts as a feedback mechanism that regulates translation of the *Preproinsulin2* (*Ins2)* mRNA (Lee et al., 2012). Rodents encode two *prepronsulin* (*Ins*) genes; however, interaction between HuD and the *preproinsulin1* (*Ins1*) transcript was not reported. Insulin is secreted from β cells in response to high levels of glucose. Circulating insulin can then bind the insulin receptor (INSR) on the surface of β cells and, through the PI3K/AKT pathway, the transcriptional repressor FOXO1 is phosphorylated. Phosphorylation of FOXO1 de-represses transcription of HuD. The HuD protein then binds the 5′UTR of *Ins2* mRNA and decreases *Ins2* translation, maintaining plasma insulin homeostasis. Consistently, HuD^–/–^ mice displayed higher insulin levels and improved glucose tolerance, whereas transgenic mice overexpressing HuD had lower insulin levels and were glucose intolerant, reportedly due to less readily releasable insulin pools (Kim et al., 2014). It has also been demonstrated that nuclear HuD is increased under ER stress resulting in decreased intracellular insulin biosynthesis and decreased plasma insulin homeostasis (Yoo, 2013). In addition to regulating *Ins2* translation, HuD also regulates the translation of two genes encoding proteins important for β cell survival in stress conditions. Autophagy-related Gene 5 (ATG5) is a protein that can mediate stress induced β cell death (Fujimoto et al., 2009). HuD binds to AREs in the 3′UTR of *Atg5* and enhances the assembly of polysomes to increase ATG5 protein levels. HuD also modulates β cell function through mitochondrial dynamics and stabilizing the mitochondrial gene *Mitofusin2* (*Mfn2*), which encodes a protein which mediates mitochondrial fusion and metabolism, and is an inhibitor of apoptosis in β cells (Baltrusch, 2016; Hong et al., 2020). Taken together, it is clear that RNA regulation by HuD is required for proper function and survival of pancreatic β cells through multiple mechanisms and pathways.

#### Polypyrimidine-Tract-Binding Protein (hnRNP1/PTB/PTBP1)

The polypyrimidine-tract-binding proteins (PTBs) are a group of RBPs that function through binding-mediated modifications in target mRNA (Wollerton et al., 2001; Mitchell et al., 2003; Auweter et al., 2007) to either recruit or block other trans-acting factors. PTB has four RRM domains each with specific consensus binding sequences that all bind stretches of pyrimidines (Sawicka et al., 2008). Additionally, PTBs can shuttle between the nucleus and the cytoplasm (Perez et al., 1997; Kamath et al., 2001; Li and Yen, 2002). PTB proteins have been implicated in the regulation of several RNA metabolism events including alternative splicing (Garcia-Blanco et al., 1989), polyadenylation (Lou et al., 1999; Castelo-Branco et al., 2004), mRNA stability (Wollerton et al., 2004), and translation (Jang and Wimmer, 1990). In the pancreas, PTB proteins have been shown to regulate insulin mRNA (human *INS* and rodent *Ins1* and *Ins2*) (Tillmar et al., 2002; Tillmar and Welsh, 2002; Fred et al., 2010, 2011, 2016) and insulin secretory granule biogenesis (Knoch et al., 2004, 2006). Specifically, the binding of PTB to *INS/Ins* mRNA increases in response to increased glucose and hypoxia (Tillmar et al., 2002; Tillmar and Welsh, 2002; Fred et al., 2011). Mutations in the *INS/Ins* 3′UTR or decreases in PTB expression by RNAi both result in decreased insulin and reporter expression respectively (Tillmar et al., 2002; Fred et al., 2016). Furthermore, binding of PTB to the 5′UTR of *INS* mRNA correspond to cap-independent translation of insulin mRNA (Fred et al., 2011). In addition to changes in PTB binding, T-cell restricted intracellular antigen 1-related protein (TIAR) also increases binding to *INS* mRNA during glucose stimulation (Fred et al., 2010). These proteins cooperate to regulate *INS* mRNA stability and biosynthesis (Fred et al., 2010). While cap-independent *INS* mRNA translation only accounts for a small portion of total translation, it can contribute 40–100% of insulin biosynthesis during stress conditions (Fred et al., 2011). In healthy β cells, transient increase in glucose levels increases PTB binding to 3′UTR promoting mRNA stability and 5′UTR promoting modest levels of cap-independent translation. However, prolonged high glucose exposure results in decreased PTB protein and ultimately decreased insulin biosynthesis (Fred et al., 2010). This is in part due to increased levels of miR-133a which targets PTB mRNA and could explain the mechanism for hyperglycemia-induced β cell dysfunction (Fred et al., 2010).

In addition to binding *INS/Ins* mRNA directly, PTB has been shown to bind and regulate components of the insulin secretory granules in response to changes in blood glucose levels. During glucose stimulated insulin secretion (GSIS), newly synthesized insulin granules preferentially undergo exocytosis (Gold et al., 1982; Halban, 1982) and this 2010process is impaired in T2D. New secretory granules are synthesized in response to glucose stimulation in the β cell, partially through regulation of PTB. Upon β cell stimulation (glucose or GLP-1), PTB is translocated from the nucleus to the cytoplasm (Knoch et al., 2004, 2006), where this process not only promotes stability of insulin mRNA (as described above), but also increases the stability of several insulin secretory granule proteins (Knoch et al., 2004). PTB translocation results from phosphorylation by PKA downstream of GLP-1 receptor and is cAMP-dependent (Knoch et al., 2006). The activated and cytosolic PTB is then able to bind and stabilize mRNAs that code for secretory granule proteins with putative PTB binding sites in the 3′UTR. Furthermore, knockdown of PTB by RNAi results in decreased expression of target mRNAs and secretory granules (Knoch et al., 2004). Taken together, glucose/GPL-1 dependent stimulation of β cells results in cytoplasmic translocation of PTB where it can act to stabilize insulin mRNA and components of the insulin secretory granule. In light of these findings, it is evident that PTB expression and activation represents a critical component in regulating GSIS.

#### Neuro-Oncological Ventral Antigens (NOVA1, NOVA2)

The Neuro-oncological ventral antigens (NOVA) are a family of two RBPs (NOVA1 and NOVA2) that account for approximately 700 alternative splicing events in neurons (Ule et al., 2005, 2006; Licatalosi et al., 2008; Zhang et al., 2010) and have been implicated in regulating alternative polyadenylation (Licatalosi et al., 2008). Both NOVA proteins bind to YCAY consensus sequences in target mRNAs through three hnRNPK-homology (KH)-type RNA binding motifs (Buckanovich and Darnell, 1997; Yang et al., 1998; Jensen et al., 2000). The positions of NOVA binding relative to the alternative splice site determines exon inclusion versus exclusion; exon inclusion is correlated with NOVA downstream binding (Ule et al., 2006; Zhang et al., 2010). Both *NOVA1 and NOVA2* are expressed in the pancreatic β cell (Villate et al., 2014; Juan-Mateu et al., 2017) and *in vitro* studies suggest they contribute to alternative splicing (Eizirik et al., 2012; Villate et al., 2014; Juan-Mateu et al., 2017). Knockdown of *Nova1* by RNAi in FACS-purified rat β cells resulted in changes in alternative splicing of 4961 isoforms and impaired GSIS (Villate et al., 2014). In INS-1E cells and MIN6 cells, knockdown of *Nova1* disrupts insulin secretion through changes in alternative splicing of key exocytosis factors *PLC*β*1* and *Snap25*, and decreases in voltage-dependent Ca^2+^ current (Villate et al., 2014). NOVA1 has also been shown to regulate alternative splicing of the insulin receptor (INSR), suggesting that NOVA1 is required to promote exon 11 inclusion and expression of the INSR-B form of the receptor (Villate et al., 2014). Additionally, NOVA1 has been implicated in T1D and cytokine-induced apoptosis (Eizirik et al., 2012; Villate et al., 2014). In both cytokine-treated β cells and *Nova1* knockdown β cells, apoptosis increases through the upregulation of pro-apoptotic protein, Bim (Barthson et al., 2011; Villate et al., 2014). Similarly, *Nova1* is decreased in β cells treated with cytokines (Villate et al., 2014). *Bim* is regulated by FOXO3a, however phosphorylation of FOXO3a inhibits it’s function (Sunters et al., 2003; Zhang et al., 2011). In *Nova1* knockdown β cells, *FoxO3a* expression is increased but phosphorylation is decreased, allowing for the subsequent upregulation of *Bim*. Similarly, NOVA2 has been shown to regulate β cell survival. *NOVA2/Nova2* knockdown in INS-1E, EndoC-βH1, and sorted rat β cells resulted in increased apoptosis (Juan-Mateu et al., 2017). Together these studies have identified several roles for NOVA RBPs in the function and survival of pancreatic β cells.

#### RNA Binding FOX Homologue (RBFOX1, RBFOX2, RBFOX3)

The RBFOX family of RBPs contains three highly conserved members – RBFOX1, RBFOX2, and RBFOX3. RBFOX RBPs all contain an RRM that recognizes the specific (U)GCAUG sequence in target mRNAs to promote alterative splicing and other RNA metabolic functions (Jin et al., 2003; Ponthier et al., 2006). *Rbfox2* is nearly ubiquitously expressed across cell types and throughout development, whereas *Rbfox1* and *Rbfox3* are considerably more cell type specific or only transiently expressed. The functions of the RBFOX proteins have been studied primarily in neurons and muscle tissue, and their activity is often required for development and maturation of these cell types (Gehman et al., 2012; Wei et al., 2015; Jacko et al., 2018). During pancreas development, scRNA-Seq reveals that *Rbfox2* is detectable throughout the embryonic (E15.5 and E18.5) mouse pancreas and hESC-derived pancreatic endocrine cells (Krentz et al., 2018). This dataset also shows that *Rbfox3* appears to be transiently expressed specifically within the Neurog3^+^ endocrine progenitor population at E15.5 (Krentz et al., 2018). *Rbfox1* is not detectable in embryonic E15.5 or E18.5 mouse pancreas or within the hESC-derived endocrine cells (Krentz et al., 2018). Similarly in the adult pancreas, RNA-Seq on intact islets revealed high expression of *Rbfox2*; whereas *Rbfox1* and *Rbfox3* were barely detectable. Further analysis of the individual sorted mouse endocrine cells determined that *Rbfox2* is expressed in α, β, and δ cells; with its highest expression in the β cell population (DiGruccio et al., 2016). Within this dataset, Rbfox1 is undetectable in any of the endocrine populations, whereas Rbfox3 expression can be found in δ cells. Consistently, scRNA-Seq in adult islets show that the majority of endocrine cells express *Rbfox2*, whereas *Rbfox3* is predominantly restricted to δ cells and Rbfox1 is undetectable (DiGruccio et al., 2016; The Tabula Muris Consortium et al., 2018). Consistent with the mouse studies, bulk sequencing of human islets and other human tissues identified expression of both *RBFOX2* and *RBFOX3*, but not *RBFOX1* in whole islets (Juan-Mateu et al., 2017). Paradoxically, this group proceeded to knockdown *Rbfox2* and *Rbfox1* in rat INS1-E cells and suggested that both proteins regulate insulin content and insulin secretion through the alternative splicing of genes involved in actin regulation (Juan-Mateu et al., 2017). It is possible that there will be redundant functions of the highly conserved Rbfox family of RBPs in endocrine cells; however, it remains to be determined how their coordinated, transient and/or compensatory expression impacts endocrine cell development and function in disease states.

#### Serine/Arginine (SR)-Rich Proteins (SRSF1, SRSF3, SRSF6)

The serine/arginine (SR)-rich proteins are a large family of RBPs characterized by their serine/arginine rich domain and an RRM (Shepard and Hertel, 2009). SR proteins are involved in several aspects of RNA metabolism including both constitutive and alternative splicing events (Zhou and Fu, 2013). SR proteins can interact with core components of the spliceosome (U1 and U2 snRNPs) to promote or inhibit splice site usage. Additionally, SR proteins function in regulating mRNA transport and translation (Zhong et al., 2009). While the function of SR proteins in other cell types and systems have been reviewed extensively (Shepard and Hertel, 2009; Zhong et al., 2009; Zhou and Fu, 2013), relatively little is known about their role in β cells.

Nearly all members of the SR protein family are expressed in the mouse pancreas (The Tabula Muris Consortium et al., 2018) and several of these SR proteins become dysregulated in diabetes (Jeffery et al., 2019). In pancreatic endocrine cells, several SRSF proteins interact with the long non-coding RNA *Paupar* to influence the alternative splicing of *Pax6* to confer differential genomic binding of the PAX6 transcription factor (Kiselev et al., 2012; Singer et al., 2019). β cells express a higher ratio of the shorter PAX6 isoform lacking the 5a exon while α cells predominantly express the longer PAX6 5a isoform (Singer et al., 2019). Together, the differential expression in healthy vs. diabetic endocrine cells and function of SR proteins in mediating transcription factor function make SR proteins an interesting candidate for evaluating the role of RBPs in the onset of diabetes. Of note, the only functional studies of this family of RBPs have been on SRSF3 and many of the SR proteins themselves undergo cell type specific and stress induced alternative splicing, leaving extensive opportunity to evaluate their β cell specific functions in healthy and diabetic states.

### Changes in RNA Regulation During Diabetes

Until recently, attempts to identify the genetic causes of T1D and T2D have predominantly relied on GWAS to identify single nucleotide polymorphisms (SNPs) and associated gene expression changes that could contribute to disease. Although these approaches have successfully identified a number of causative candidate alleles, they overlook altered splicing events that may affect gene function rather than expression levels. Furthermore, it is now apparent that differences in co- and posttranscriptional processing such as alternative splicing and N6-methyladenosine (m6A) modifications, can more effectively differentiate between T2D β cells than transcriptomics alone (De Jesus et al., 2019).

### Alternative Splicing

Messenger RNA splicing occurs co-transcriptionally to remove introns from the pre-mRNA. This process is carried out by the spliceosome and is coordinated by a series of RBPs. In addition to removing introns, splicing machinery can also vary mRNAs through alternative exon and splice site usage, referred to as alternative splicing. Over 90% of human genes undergo alternative splicing, which more than quadruples the number of potential gene products (Fairbrother et al., 2002; Johnson et al., 2003). Recent work comparing transcriptomes of T2D diabetic and healthy donors identified dysregulation of 26% of alternative splicing events (Jeffery et al., 2019). The highest proportion of alternatively spliced genes in this study function in gene regulation. For example, the authors observed dysregulation of the alternative splicing regulators such as SRSF RBPs, which could each regulate hundreds of splicing events within the β cell. Stress induction of the EndoC-βH1 β cell line supported findings from human diabetic islets, showing a decrease in splicing regulators. Moreover, removal of the stress restored splicing factor expression and changes in transcriptome wide splicing events. Additionally, in a model of T1D, cytokine exposure of FAC sorted rat β cells resulted in differential expression of more than 20 RBPs involved in alternative splicing and changes in alternative splicing of cytokine regulated genes (Ortis et al., 2010). These groups all suggest that changes in the splicing landscape as well as changes in β cell differentiation markers, may be a mechanism of stress response to avoid apoptosis during the onset of diabetes, making the study of alternative splicing regulation in the β cell critical for not only understanding the pathogenesis of the disease but also in designing innovative treatment plans.

The onset of T2D and accelerated dysfunction of β cells has also been attributed to many environmental factors, including the disruption of circadian sleep/wake cycles (Gale et al., 2011). Although the disruption of circadian sleep/wake cycles has traditionally been associated with mRNA oscillations, a recent study implicates the RBP Thyroid Hormone Receptor-Associated Protein3 (THRAP3) as a regulator of alternative-splicing. This study demonstrated that THRAP3 regulated circadian clock-dependent alternative splicing by binding to and regulating alternative splicing of key exocytosis factors (Marcheva et al., 2020).

In addition to transcriptome wide changes in alternative splicing, several groups have explored the alternative splicing of specific genes involved in β cell development, function, and survival. Of note, several Maturity Diabetes of the Youth (MODY) and T2D associated genes such as *HNF-1*α, *GCK*, and *TCF7L2* have splicing variants (Prokunina-Olsson et al., 2009). While *TCF7L2* isoforms are not significantly altered in T2D (Prokunina-Olsson et al., 2009), both *HNF-1*α and *GCK* diabetes associated alleles result in alternative splicing variation (Cappelli et al., 2009; Lorini and D’Annunzio, 2009), and isoforms of HNF-1α are associated with differential efficiency in insulin gene regulation (Harries et al., 2006; Cappelli et al., 2009). T2D associated alterations in alternative splicing of *HNF-1*α and several other β cell genes are also reviewed by Dlamini et al. (2017). Based on recent findings in large transcriptomic analyses, this field is likely to grow dramatically.

### m6A RNA Methylation

N6-methyladenosine (m6A) methylation is one of the most prevalent post-transcriptional RNA modifications and is regulated by a set of RNA-binding proteins – writers, readers, and erasers (Figure 1; Zhang et al., 2019). These modifications are introduced by a group of specialized methyltransferases (“writers”) including METTL3 and METTL14. The m6A modifications can confer differential stability, changes in alternative splicing, subcellular localization, and translation efficiency (Zhang et al., 2019). The m6A modifications are often bound by a group of RBPs called readers to perform these differential functions. Finally, a third group of RBPs, including FTO (Fat Mass and Obesity-associated gene), are referred to as erasers and can remove m6A marks. A recent review by Zhang et al. (2019) details the current knowledge on mechanism and function of m6A methylation.

With respect to the β cell, a recent publication by De Jesus et al. (2019) describes the differential m6A methylation observed in T2D. RNA-Seq analysis and fluorescent labeling of human T2D islets compared to healthy controls revealed differential expression of key m6A modulators (METTL3, METTL14, ALKBH5, and YTHDF1). This finding was supported by an independent study showing decreased Mettl3/14 expression in diabetic db/db mice and type 2 diabetes patients (Wang et al., 2020). The decreased expression of m6A writers (METTL3 and METTL14) resulted in differential methylation of 6,078 sites in 4,155 genes (FDR < 0.05). The hypomethylation of genes in the T2D islets are associated with cell-cycle progression, insulin secretion, and the insulin/IGF-AKT-PDX1 pathway. This study also replicated T2D phenotypes and the m6A methylome in METTL3 or METTL14 deficient EndoC-βH1 cells and Mettl14 β-cell-specific knockout mice. Another group generated a similar β cell specific knockout of *Mettl14* in mice and observed that these mice display glucose intolerance, decreased GSIS and decreased β cell mass due to β cell death under normal conditions (Liu et al., 2019). These phenotypes are exaggerated in mice fed a high fat diet (Liu et al., 2019). Together these studies indicate a role for METTL14 and m6A modifications in the function and survival of β cells.

In the context of pancreas development, m6A writers (METTL3/14) are critical for β cell expansion and maturation but appear to be dispensable for the differentiation and maturation of other endocrine cell types (Wang et al., 2020). Wang et al. (2020) also showed that in the developing pancreas loss of *Mettl3* or *Mettl14* from endocrine progenitors independently results in hyperglycemia around weaning, but that loss of both methyltransferases results in significant hyperglycemia and hypo-insulinemia by 2-weeks of age. This functional defect is in part due to decreased proliferation and increased cell death, similar to what had been observed in the previously described β cell specific knockout mice. Additionally, using RNA-Seq and m6A Me-RIP-Seq, the authors concluded that *Mettl3/14* directly regulates the β cell maturation factor, MAFA, to promote stability. Other groups have also studied the effects of m6A modifications, particularly in adipogenesis, that could contribute to the pathogenesis of T2D (Gerken et al., 2007; Chu et al., 2008; Ben-Haim et al., 2015; Shen et al., 2015; Wood et al., 2016).

As a counterpoint to the m6A writers METTL3/14, FTO is an m6A eraser. FTO is expressed in a variety of cell and tissue types including endocrine cells (Taneera et al., 2015; DiGruccio et al., 2016; The Tabula Muris Consortium et al., 2018). Whole body knockouts and nervous system specific knockouts of *Fto* in mice result in postnatal growth deficiencies (Gao et al., 2010), however, its specific function in β cells is debated. In T2D human islets, FTO expression is reduced (Kirkpatrick et al., 2010; Taneera et al., 2018) and the FTO gene has decreased DNA methylation (Dayeh et al., 2014). Several groups have investigated the functional role of FTO in pancreatic islets using different experimental models. Overexpression of FTO in rat INS-1 cells appeared to affect first wave insulin secretion (Russell and Morgan, 2011), whereas overexpression in mouse MIN6 cells resulted in the inhibition of GSIS without changes in insulin gene expression (Fan et al., 2015). Given the discrepant results of these studies, in addition to the caveats associated with overexpression studies, perhaps the more relevant functional assessment was the use of siRNA knockdown to deplete FTO in an engineered human insulin secretion reporter rat β cell line (GRINCH) (Taneera et al., 2018). In these experiments, depletion of FTO resulted in decreased insulin mRNA expression and insulin secretion (Taneera et al., 2018).

While these emerging studies are beginning to highlight the relevance of m6A RNA methylation in β cell function, and the potential contribution of alterations in this RNA modification to the onset of diabetes, the underlying mechanism and direct targets of m6A methylation and demethylation that contribute to the observed functional changes have yet to be determined. Future studies of the islet cell-specific changes in RNA modifications that result from defects in the m6A pathway will provide critical new information about the regulation of islet function in normal and disease conditions.

## Discussion

Traditionally, endocrine cells have been molecularly defined by their cell-specific transcriptomes. Furthermore, validation of human diabetes GWAS studies have relied primarily on gene expression changes associated with disease. More recently, however, large scale high-throughput sequencing efforts have revealed the previously unappreciated importance of co- and post-transcriptional RNA regulation in the specification and function of differentiated cells. These high-resolution sequencing technologies have not only identified changes in expression levels, they have unveiled numerous RNA modifications and alternative splicing events within each of the islet endocrine cell populations and in individual β cells. This discovery has prompted investigation into the many RBPs that are expressed in the pancreatic islet and those that become dysregulated in β cells undergoing stress conditions that mimic diabetes (Table 1). Each of these RBPs can have hundreds of targets and affect multiple pathways within a cell in both physiological and pathophysiological conditions (Keene, 2007; Hogan et al., 2008; Lukong et al., 2008; Blanchette et al., 2009; Li et al., 2014), making their potential impact on cellular identity and function pervasive. This also implies that there are many more layers of regulation in the β cell, particularly mediated by RBPs, that have yet to be explored. The knowledge gained from understanding regulation of β cell development and function at the level of mRNA modifications could be immensely useful to optimize protocols to generate insulin-secreting β-like cells from human stem cells (Hrvatin et al., 2014), which is currently a promising method of replacing β cell loss in T1D. Furthermore, characterization of dysregulated splicing events could open therapeutic opportunities to correct specific mRNAs using antisense oligonucleotide (ASO) technologies. ASOs are small synthetic nucleotide sequences that can target specific mRNA transcripts to target and eliminate anomalous splice variants (Schoch and Miller, 2017). Treatment with ASOs has become increasingly promising for treating neurodegenerative diseases (Schoch and Miller, 2017) and could be an innovative mechanism to correct aberrant splicing defects occurring in T2D β cells. Overall, the study of RBPs and RNA modifications are primed to be the next frontier of mechanisms that regulate β cell formation, function, and in the development of novel therapeutics.

## Author Contributions

NM researched the literature and wrote the manuscript. LS researched the literature and edited the manuscript. Both authors contributed to the article and approved the submitted version.

## Conflict of Interest

The authors declare that the research was conducted in the absence of any commercial or financial relationships that could be construed as a potential conflict of interest.
